# Nipple-Sparing Mastectomy and Prepectoral Implant/Acellular Dermal Matrix Wrap Reconstruction in Large Ptotic Breasts

**DOI:** 10.1097/GOX.0000000000002289

**Published:** 2019-07-25

**Authors:** Haitham H. Khalil, Marco N. Malahias, Sherif Youssif, Tarek Ashour, Saif Rhobaye, Tahir Faroq

**Affiliations:** From the * Plastic and Reconstructive Surgery Division, Good Hope Hospital, University Hospitals Birmingham Trust, Birmingham, United Kingdom; † Department of Plastic and Reconstructive Surgery, Cairo University Hospitals, Cairo, Egypt; ‡ Department of Breast Surgery, Good Hope Hospital, University Hospitals Birmingham Trust, Birmingham, United Kingdom.

## Abstract

Nipple-sparing mastectomy (NSM) with simultaneous prepectoral direct to implant reconstruction and acellular dermal matrix (ADM) is increasingly offered to patients opting for prophylactic or therapeutic mastectomies. The recent introduction of prepectoral implant/ADM in the armamentarium of breast reconstruction has proven to reduce pain and animation deformity. Despite this promising method, patients with macromastia and ptotic breasts remain a challenging group to treat. More often they would require secondary corrective procedures and can experience high failure rate and unsatisfactory outcomes. The authors present their experience in utilizing a bipedicled nipple-areola complex dermal flap through Wise pattern to achieve a successful NSM with prepectoral implant/ADM (Braxon) wrap (IBW) as a single stage in patients with large ptotic breasts. Patients seeking NSM with large ptotic breasts were included in the study in the period between 2016 and 2018. They were offered a single-stage Wise pattern NSM and immediate prepectoral IBW breast reconstruction. The technique and outcomes were recorded. Sixteen reconstructions were performed in 8 women with a median age of 32 years (range, 27–50 years) and a median body mass index of 32 kg/m^2^ (range, 29–39 kg/m^2^). The resected breasts weight ranged from 750 to 1,600 g (median, 890 g). All procedures were completed successfully with no nipple-areola complex necrosis or failure experienced during the follow-up period (range, 3–24 months) with all patients reported excellent satisfaction. The authors’ results demonstrate that this technique could be safely planned for NSM in large ptotic breasts with excellent durable outcomes.

## Abstract 2

Supplemental Digital Content is available in the text.

Nipple-sparing mastectomy (NSM) in patients with significant ptosis and macromastia poses a substantial challenge to the nipple-areola complex (NAC) and breast skin envelope vascularity.^1^ However, a principle fact illustrated in previous studies demonstrated that the NAC blood supply could be maintained by the subdermal plexus.^2^ Notably, the tenets of NSM have been well established in literature gaining more popularity driven by high level of patient satisfaction.^3–6^ Nonetheless, in some patients, considerable breast ptosis could limit the satisfactory aesthetic result. Respectively, previous approaches have been described to facilitate NSM with either staged or simultaneous mastopexy for breast reconstruction in large ptotic breasts.^7–18^. From a different perspective, the resurgence of prepectoral prosthesis/acellular dermal matrix (ADM) breast reconstruction has provided numerous advantages with superior aesthetic outcomes.^19–24^

The authors present a series of Wise pattern NSM using bipedicled NAC dermal flaps technique with immediate prepectoral Implant/ADM Braxon Wrap (IBW) breast reconstruction as a single stage which to their best knowledge would be the first to be reported in this context.

## PATIENTS AND METHODS

**Fig. 1.**
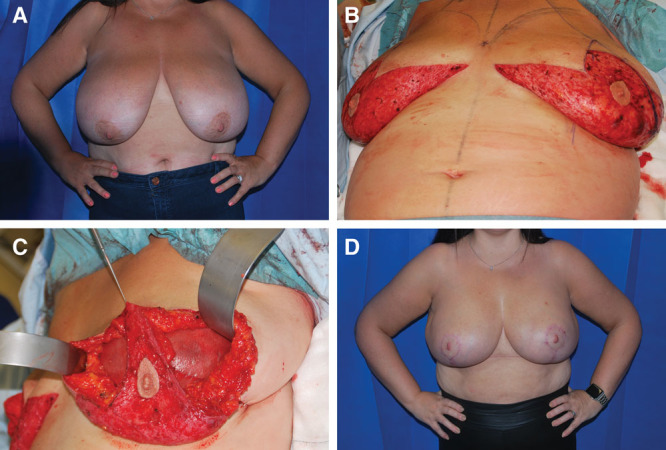
Bilateral prepectoral IBW breast reconstruction post risk reducing mastectomies in large ptotic breasts. A, Forty-two year old lady with macromastia and BRCA 1 gene mutation with a BMI 39 considered for bilateral Wise pattern NSM and immediate prepectoral IBW. Of note the NAC-sternal notch distance is 34 cm. B, Combined approach for breast and plastic surgical team through which standard Wise pattern reduction mammoplasty technique is performed with isolation of the NAC and de-epithelization of the lower breast pole. A vertical incision (8–10 cm) is performed at the lateral breast pillar (blue marking) within the de-epithelized zone through which the mastectomy is performed. C, Intraoperative photograph postcompletion of prophylactic NSM (breast weight 1,600 g each breast) demonstrating the superior and inferior dermal NAC (bipedicled) flaps. The IBW is secured in the breast pocket with PDS sutures laterally and superiorly. Note the meticulous de-epithelization of both superior and inferior NAC flaps to avoid any damage to the subdermal vascular plexus which supplies the NAC. Note that mastectomy flaps viability was assessed through examining the undersurface for preservation of the subcutaneous fat to protect the subdermal plexus, in addition to adequate bleeding through refreshing of the wound edges and examination of the skin surface capillary refill. Any doubt in the viability of the mastectomy flaps should be a trigger to conversion to expander based or defer reconstruction to second stage. D, Postoperative photograph 6 months showing completely healthy NACs and scars healed with primary intention providing good shape and symmetry of both breasts.

Prospectively collected data from 33 patients who underwent 48 consecutive prepectoral implant/expander-ADM Braxon wrap (Decomed S.r.l, Venezia, Italy) breast reconstructions in the period between July 2016 and November 2018 were retrospectively reviewed. Within this cohort, 8 patients (16 reconstructions) who underwent bilateral Wise pattern NSM were identified and will be the focus of this series. The indication included risk reducing mastectomies (RRM) with exclusion of high-risk patients (previous breast surgery, local radiotherapy, and general risk factors as diabetes and active smoking), while obesity alone was not merely considered as an exclusion factor. All patients were managed through a breast oncoplastic multidisciplinary team including breast surgeons and reconstructive surgeon (H.H.K.). This included preoperative preparation and radiological investigations to exclude any breast pathology. The surgical technique of NSM with Wise pattern mastopexy and prepectoral IBW reconstruction is demonstrated in Figure 1 and Supplemental Digital Content 1 and 2 (**see figure, Supplemental Digital Content 1**, which displays a medical illustration of surgical technique, **http://links.lww.com/PRSGO/B139**) (See Video, [online], which demonstrates the intraoperative surgical technique of NSM with simultaneous Wise pattern mastopexy and immediate prepectoral IBW breast reconstruction. Patient satisfaction survey was conducted during clinical follow-up.

**Video 1.** This video demonstrates the intraoperative surgical technique of NSM with simultaneous Wise pattern mastopexy and immediate prepectoral IBW breast reconstruction.

## RESULTS

The median age for these 8 patients was 32 years (range, 27–50 years) with a median body mass index (BMI) of 32 kg/m^2^ (range, 29–39 kg/m^2^). All procedures were completed successfully using Mentor round implants 450–600 cc (median, 550 cc) (Fig. 1). The resected breast tissue weight ranged from 750 to 1,600 g (median, 890 g). One patient developed congestion of 1 NAC intraoperatively postwound closure. This was successfully managed by loose suturing of the areola and application of Incisional Negative Pressure Wound Therapy (Prevena dressing; Kinetic Concepts, Inc, San Antonio, Tex.) followed by delayed primary closure 1 week later (**see figure, Supplemental Digital Content 2**, which displays an (a) intraoperative photograph demonstrating a patient who underwent bilateral prophylactic Wise pattern NSM and immediate prepectoral IBW, **http://links.lww.com/PRSGO/B140**). During the follow-up period of 3–24 months (median, 12 months), no NAC necrosis, seroma, late failure, or revisional corrective surgery was experienced with an overall excellent satisfaction.

## DISCUSSION

Macromastia is often considered a relative contraindication for NSM due to the possible increased risk of wound and NAC ischemic complications, malposition, and possible overall failure.^8,25–27^ Furthermore, factors including breast weight over 1,000 g, prepectoral reconstruction, and BMI over 35 would significantly accelerate these outcomes.^18,28^ Nevertheless, several studies have reported NSM in large ptotic breasts with either staged^7–10^ or simultaneous mastopexy for autologous and submuscular implant breast reconstructions.^11–18^ It is noteworthy that previous studies demonstrated high complication rate, suboptimal aesthetic results which has subsequently demanded secondary revisional procedures^8,25–27,29,30^ with partial and total NAC loss ranging from 0% to 18.7%.^1^ We advocated meticulous intraoperatively assessment of the mastectomy flaps for quality and viability particularly due to the overstretched quality of native skin of large ptotic breast. This was through identifying bleeding edges and well-preserved fatty layer on their undersurface to protect the subdermal plexus. There is no evidence that removing the subcutaneous fat in the mastectomy flap actually increases the disease free or the survival so meticulous dissection in the distinct anatomical plane is paramount for successful outcomes.^21^ In essence, close collaboration between breast and oncoplastic surgeons to achieve the most optimal risk reduction or oncological outcome along with the best aesthetic results is imperative. The introduction of bioengineered ADM has expedited the resurgence of prepectoral reconstruction^20,31^ with significant improvement when compared with the historically poor outcomes in the 1970s.^1^ Along these lines, we have used the prepectoral IBW as it is less invasive, less operative time, and more cost-effective as only 1 sheet is utilized with reduction in capsular contraction which is in accordance with previously published series.^32,33^ Acknowledging the fact that immediate implant reconstruction exerts greater stress on mastectomy skin flaps therefore increasing risks of ischemic complications,^18,27^ we have attempted to alleviate this effect through anchoring the IBW superiorly to the chest wall. Simultaneous reshaping of the breast skin envelope allows the direct opposition of the skin to the ADM hence eliminating the dead space and decreasing the risk of seroma which has also been supported by previous studies.^11,34^ Contrarily, correcting breast ptosis over an implant with a delayed procedure can be difficult due to underlying scarring and formed capsule.^11^ Emphasis on patient selection criteria is imperative for overall success notably performed for prophylactic reasons rather than therapeutic. This is due to the fact that complications rates with simultaneous mastopexy are likely to be higher when performed in therapeutic setting.^11,35^ Interestingly, high BMI in this series did not contribute to an increase to the overall complication risks. In addition to the well-established indications for Incisional Negative Pressure Wound Therapy reported in the literature,^36^ we have extended this to manage a congested NAC successfully with full recovery. Although the most significant finding in this series is that all 16 reconstructions were completed without any case of NAC necrosis, there is limitation to this study due to its retrospective nature, small sample size, and the relatively short follow-up period.

## CONCLUSIONS

To our knowledge, this current series is the first to demonstrate the outcomes of NSM using a bipedicled NAC Wise pattern technique in large ptotic breasts and immediate prepectoral IBW breast reconstruction as a single stage. The short-term results have shown that this approach is safe and durable, with improved outcomes and low complication rate.

## ACKNOWLEDGMENTS

We thank Jamie Ryan-Ainslie, Heidi Twaites, and the entire Medical Illustration Team at University Hospitals Birmingham (UHB) for their expert input.

## Supplementary Material
