# Supplementary figures and images for: Nipple-Sparing Mastectomy and Prepectoral Implant/Acellular Dermal Matrix Wrap Reconstruction in Large Ptotic Breasts
Source: Plast Reconstr Surg Glob Open. 2019 Jul 25;7(7):e2289. doi: 10.1097/GOX.0000000000002289 (PMC6952144; doi:10.1097/GOX.0000000000002289)

**A**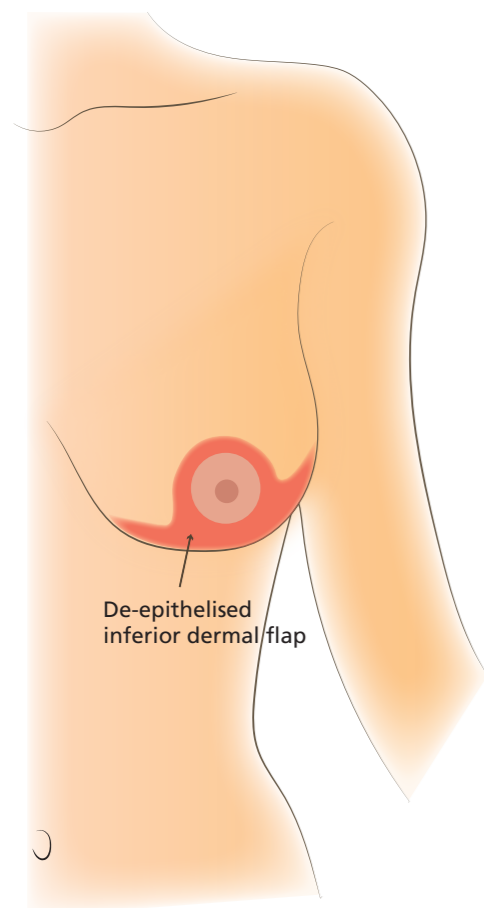**B**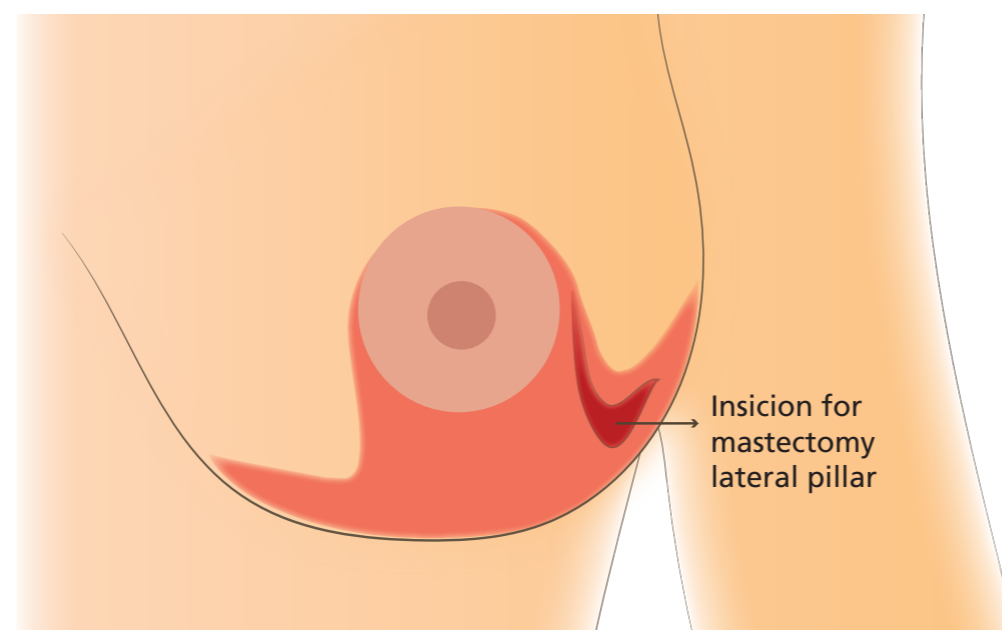**C**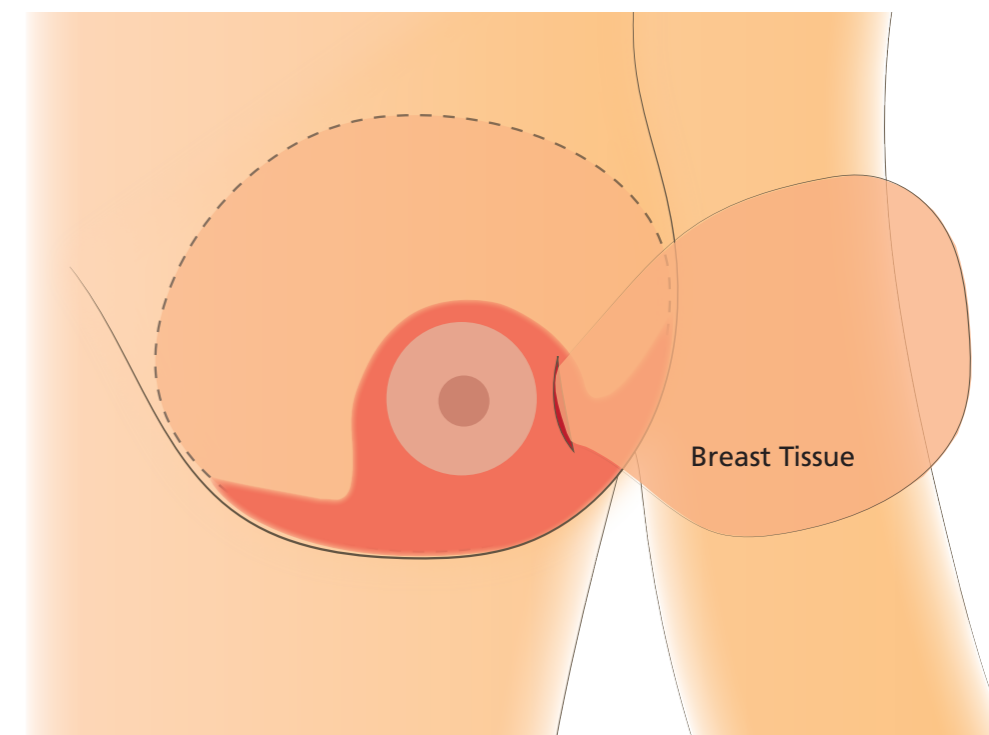**D**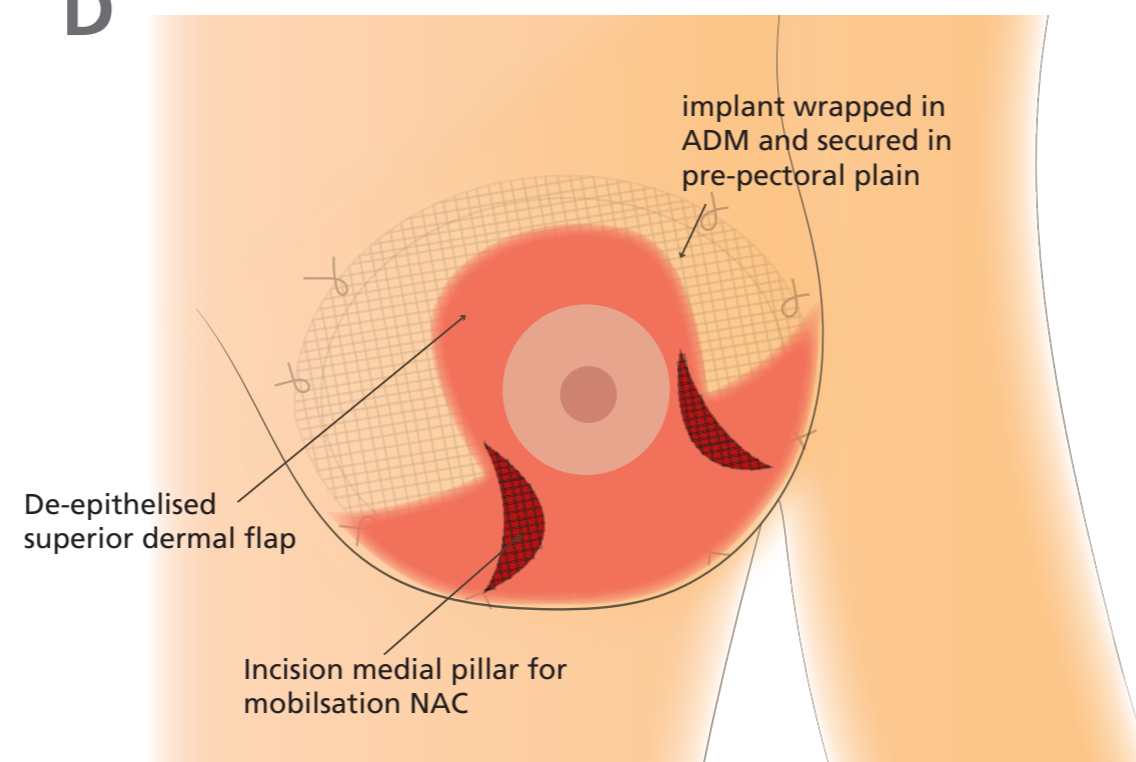**E**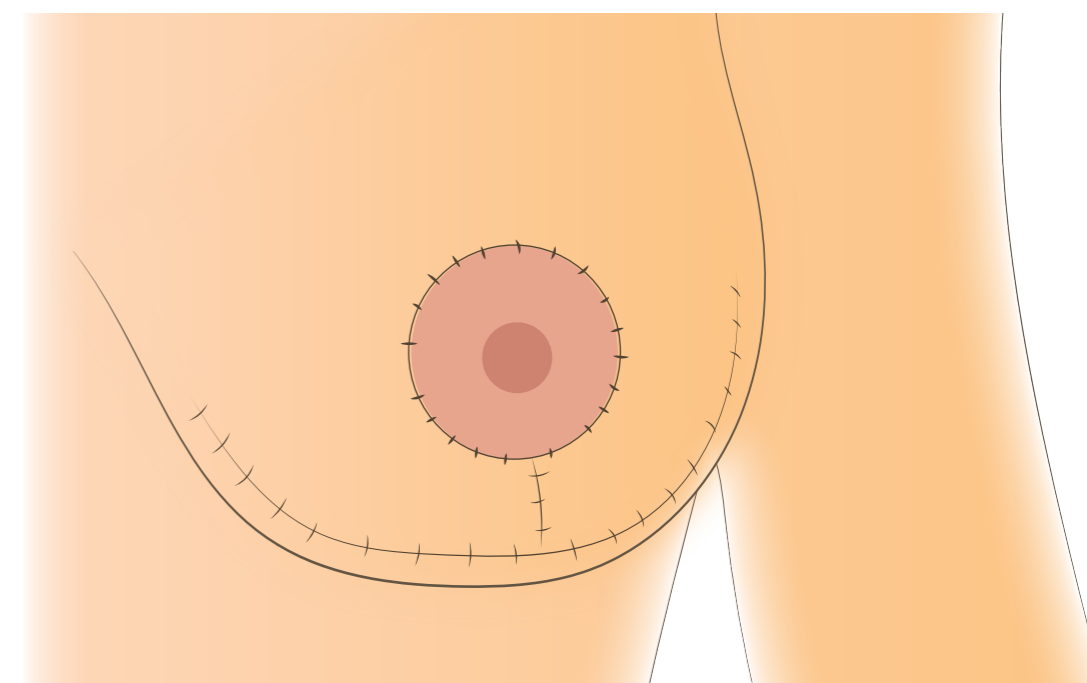

*[Signature]*  
13 09 18

Supplement: Supplementary file 2 [file gox-7-e2289-s002.pdf]

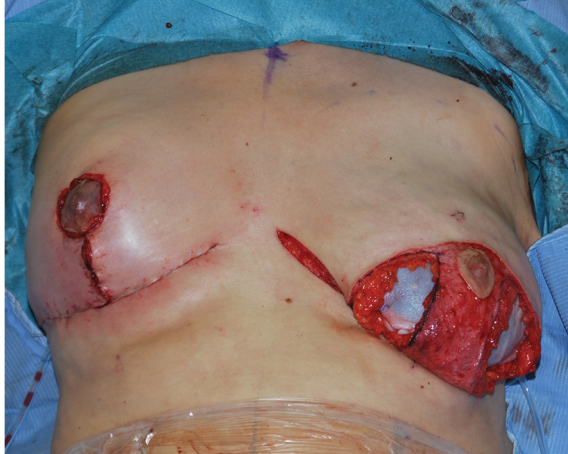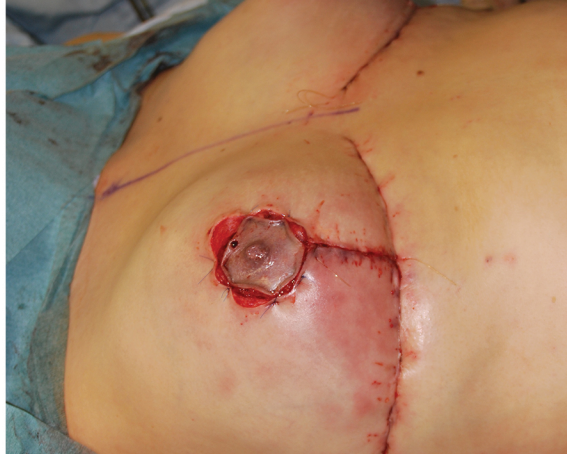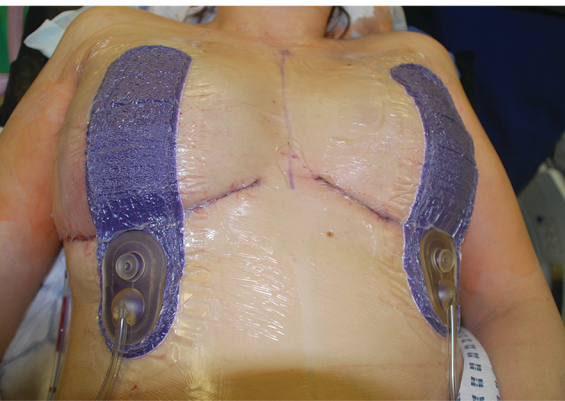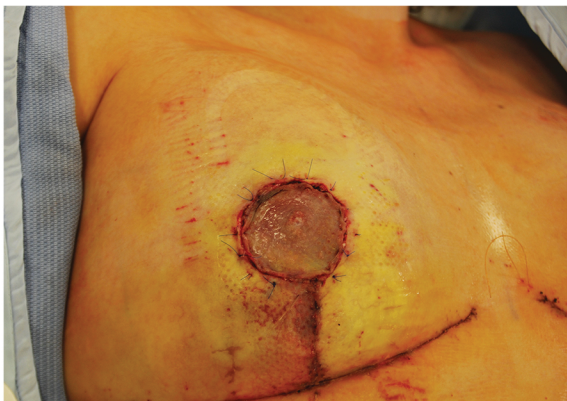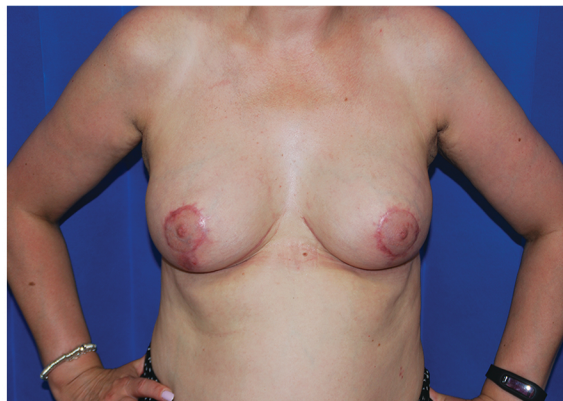

Supplement: Supplementary file 3 [file gox-7-e2289-s003.pdf]
